# The alleviating effect of exogenous polyamines on heat stress susceptibility of different heat resistant wheat (*Triticum aestivum* L.) varieties

**DOI:** 10.1038/s41598-020-64468-5

**Published:** 2020-05-04

**Authors:** Jianguo Jing, Suyan Guo, Youfang Li, Weihua Li

**Affiliations:** Key Laboratory of Oasis Eco-Agriculture, Xinjiang Production and Construction, Corps, Shihezi University, 832003 Shihezi, Xinjiang China

**Keywords:** Abiotic, Abiotic, Abiotic, Abiotic, Heat, Heat, Heat, Heat

## Abstract

High temperature inhibits wheat grain filling. Polyamines (PAs) are closely associated with plant resistance caused by abiotic stress. However, little is known about the effect of PAs on the grain filling of wheat under heat stress. Two wheat varieties differing in heat resistance were used, and endogenous PAs levels were measured during grain filling under normal growth conditions outside the greenhouse (CK), artificially simulated high temperature (HT), artificially simulated high temperature plus exogenous application of spermine (HT + Spm) and artificially simulated high temperature plus spermidine (HT + Spd) treatments. Additionally, the variation of antioxidant enzymatic activities and osmotic adjustable substances content in grains was measured during grain filling. The results showed that compared with HT,HT + Spm and HT + Spd significantly increased grain weight of XC 6 (heat-resistant variety) by 19% and 5%, and XC 31 (heat-sensitive variety) by 31% and 34%, activity of superoxide dismutase (SOD), peroxidase (POD)and catalase (CAT) and content of Spm, Spd, and proline (Pro) increased significantly, while putrescine (Put), malondialdehyde (MDA) and soluble sugar (SS)contentdecreased during grain filling; The correlation analysis showed that grain weight was negatively correlated with the content of PUT, MDA, Pro and activity of SOD and CAT and positively correlated with the content of Spd and activity of POD in grains. Our results indicated that exogenous Spm and Spd could alleviate the heat injury of grain filling.

## Introduction

Wheat (*Triticum aestivum* L.) is one of the main food crops all over the world, and its production is directly related to the issue of food security. The optimum temperature of wheat at filling stage was 17–23 °C. When the daily maximum temperature is above 30 °C, the growth and development of wheat will be adversely affected. In severe cases, the yield decreased about 20% compared with the normal temperature^1^. In recent years, the global average temperature has been on the rise, and the frequency of high temperature is increasing, which poses a serious threat to wheat yield and quality^2,3^. Heat stress induces various biochemical and physiological responses in plants because of alteration of water content within the plant tissue and oxidative stress such as protein denaturation, lipid peroxidation, MDA accumulation and pigment degradation due to produced reactive oxygen species^4^. In the long-term evolution process, plants have formed an effective mechanism to cope with environmental stress. Accumulation of amino acids (such as Pro), sugars (such as sucrose, trehalose and sorbitol), sugar alcohols (such as manitol), and amines (such as glycine betaine and PAs) are for osmotic protection in plant to preserve water^5–7^. Meanwhile, enzymatic and non-enzymatic antioxidants were up-regulated to overcome oxidative stress^8^. The most important antioxidant enzymes are superoxide dismutase (SOD), catalase (CAT) and peroxidase (POD). SOD converts ROS into H_2_O_2_ and O_2_, while CAT and POD convert H_2_O_2_ into H_2_O^4,9^. Nevertheless, growth and development will be threatened when the stress exceeds its regulatory range^10,11^. At present, in the field of production the main way to enhance the tolerance of wheat to high temperature stressis to select heat-resistant wheat varieties through the accumulation of heat-resistant genes^12,13^, but this process takes a long time and is greatly affected by the environment^14^.

Polyamines (PAs), such as putrescine(Put), spermidine(Spd), and spermine (Spm), can be found in relatively high amounts in all living cells. They have been described as endogenous plant growth regulators or intracellular messengers that regulate plant growth, development, and responses to abiotic stresses^5,15–18^. Zhang *et al*. (2010)suggested that application of spermidine (Spd, 0.1 mM L^−1^) under condition of abiotic stress can increase the soluble protein content of leaves and reduce the relative conductivity and malondialdehyde (MDA) content;^19^ Many researches have reported that polyamine treatment under adverse conditions can maintain high chlorophyll content, promote the balance of O_2_^−^ content, reduce plasma membrane permeability, and maintain the integrity of cell plasma membrane in seedling leaves(1 mM L^–1^)^20,21^. In addition, PAs were thought to be involved in the regulation of grain development.

The polyamine concentration of aborting maize grains (*Zea mays* L.)was significantly lower than that of normal grains, and the polyamine concentration was positively correlated with the endosperm nuclei number^22^. Yang *et al*. (2008) found that higher levels of spermine (Spm, 240 nM g^–1^ FW) and Spd (300 nM g^−1^ FW) could promote grain filling and increase the grain weight of rice(*Oryza sativa* L.)^23^. Tan *et al*. (2009) showed that the low grain filling rate and low grain weight of inferior grains in super rice may be related to the fact that there are low concentrations of Spd (220 nM g^−1^ FW) and Spm (180 nM g^−1^ FW) and low Spd/Put and Spm/Put ratios in grains^24^. These studies suggest that the PAs are related to regulate the grain development in plants. However, little is known about the relationship among PAs, antioxidant enzymatic activities and osmotic adjustable substances content in the regulation of wheat grain filling under heat stress. The main objective of this study was to investigate the effect of heat stress on grain filling oftwo wheat varieties and we also tried to determine whether exogenous PAs could regulate the grain filling of wheat by regulating the changes of endogenous PAs under high temperature stress.

## Results

### Yield and yield components

Compared with CK, HT treatment significantly reduced grain number per panicle (GNP), thousand grain weight (TGW), grain weight per panicle (GWP) and yield of XC 6 (reduced by 18%, 13%, 25% and 29% respectively in 2018 and by 16%, 10%, 20% and 25% respectively in 2019); and HT treatment also significantly reduced GNP, TGW, GWP and yield of XC 31 by 26%, 15%, 32% and 37% respectively in 2018 (Table 1, Year, 2018) and by 10%, 6%, 30% and 16% respectively in 2019(Table 1, Year, 2019), indicating that the responses of XC6 and XC31 to high temperature stress were different in two years, XC 6 was superior to XC 31 in 2018, while XC 31 was superior to XC 6 in 2019. Moreover, the decrease of GWP and TGW caused by HT treatment in the early stage of grain-filling was the main reason for the decrease of wheat yield. Compared with HT, exogenous spraying Spm under high temperature treatment (HT + Spm) significantly increased GNP and TGW, GWP and yield of XC 6 by 17%, 7%, 19% and 25%, respectively, and XC 31by 24%, 10%, 31% and 36%, respectively in 2018 (Table 1, Year, 2018), and XC 6 by 6%, 8%,19% and 15%, respectively, and XC 31 by 8%, 2%, 26% and 10%, respectively in 2019(Table 1, Year, 2019). Compared with HT, exogenous spraying Spd under high temperature treatment (HT + Spd) significantly increased GNP, TGW, GWP and yield of XC 6by 5%, 5%, 5% and 11%, and XC 31 by 17%, 12%, 34% and 31% in 2018 (Table 1, Year, 2018), and XC 6 by 6%, 7%, 14% and 13%, respectively, and XC 31 by 8%, 1%, 21% and 9%, respectively in 2019 (Table 1, Year, 2019), indicating that exogenous Spm and Spd could significantly alleviate the damage of high temperature to wheat grain-filling under HT treatment, and the alleviating effects of two PAswere different in two years.

**Table 1:** Effects of polyamines on yield and yield components of two varieties (XC 6 and XC 31) under high temperature stress in 2018and 2019.

Year	Varieties	Treatment	Weightper panicle(g)	Grainsnumbersper panicle	Thousand grains weight (g)	Grain yield (kg hm^−2^)
2018	XC6	CK	2.29 ± 0.036^a^	45.07 ± 0.46^a^	45.92 ± 0.20^a^	5277.66 ± 76.81^a^
		HT	1.72 ± 0.017^d^	37.00 ± 0.20^d^	39.81 ± 0.29^d^	3756.17 ± 47.68^d^
		HT + Spm	2.04 ± 0.004^b^	43.33 ± 0.12^b^	42.51 ± 0.15^b^	4697.74 ± 26.94^b^
		HT + Spd	1.80 ± 0.012^c^	39.00 ± 0.20^c^	41.81 ± 0.37^c^	4158.38 ± 48.54^c^
	XC31	CK	2.05 ± 0.087^a^	48.33 ± 0.31^a^	46.04 ± 0.25^a^	5674.07 ± 54.98^a^
		HT	1.40 ± 0.003^c^	35.87 ± 0.42^c^	39.18 ± 0.34^d^	3583.80 ± 61.07^d^
		HT + Spm	1.83 ± 0.030^b^	44.33 ± 0.42^b^	43.08 ± 0.13^c^	4869.91 ± 59.85^b^
		HT + Spd	1.87 ± 0.005^b^	42.07 ± 0.12^b^	43.77 ± 0.40^b^	4694.93 ± 55.33^b^
2019	XC6	CK	2.37 ± 0.014^c^	47.07 ± 0.76^a^	50.30 ± 0.54^b^	6036.73 ± 39.18^b^
		HT	1.89 ± 0.016 ^f^	39.33 ± 0.61^c^	45.17 ± 0.15^d^	4530.89 ± 72.96^d^
		CK + Spm	2.52 ± 0.012^a^	47.93 ± 0.83^a^	52.43 ± 0.85^a^	6408.27 ± 103.01^a^
		CK + Spd	2.46 ± 0.009^b^	47.60 ± 0.20^a^	50.60 ± 0.24^b^	6141.51 ± 55.03^b^
		HT + Spm	2.24 ± 0.041^d^	41.80 ± 0.87^b^	48.84 ± 0.29^c^	5205.49 ± 83.68^c^
		HT + Spd	2.15 ± 0.021^e^	41.73 ± 0.58^b^	48.31 ± 0.07^c^	5141.22 ± 78.58^c^
	XC31	CK	2.05 ± 0.037^b^	43.07 ± 0.42^c^	45.80 ± 0.15^a^	5029.73 ± 46.44^b^
		HT	1.44 ± 0.005^d^	38.60 ± 0.20^e^	42.97 ± 0.29^c^	4229.24 ± 40.88^d^
		CK + Spm	2.23 ± 0.022^a^	45.87 ± 0.23^a^	46.11 ± 0.30^a^	5393.46 ± 51.83^a^
		CK + Spd	2.17 ± 0.088^a^	43.73 ± 0.12^b^	45.89 ± 0.69^a^	5117.16 ± 65.33^b^
		HT + Spm	1.82 ± 0.004^c^	41.73 ± 0.46^d^	43.90 ± 0.73^b^	4672.03 ± 127.38^c^
		HT + Spd	1.74 ± 0.101^c^	41.67 ± 0.42^d^	43.54 ± 0.33^bc^	4625.89 ± 11.18^c^

Values (means±SE, n = 3) followed by different letters among different treatments are significantly different according to the Duncan’s multiple range tests (P < 0.05).

### Grain filling

**Figure 1:**
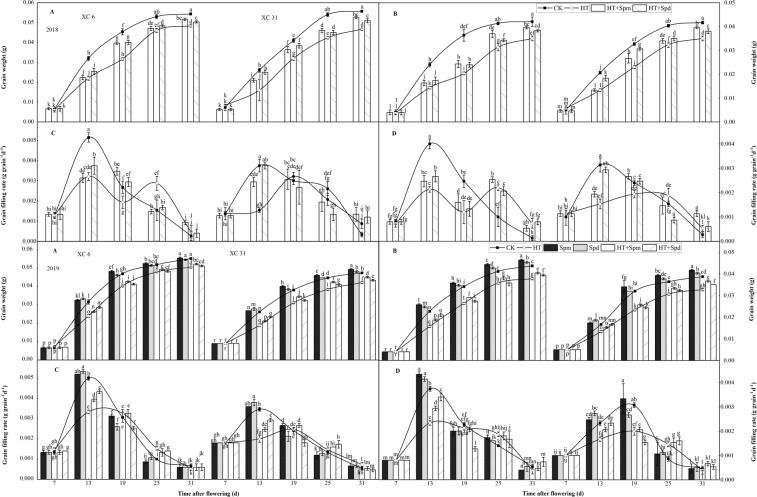
Changes of grain weightsand grain filling rates (**A–C**: superior grain and **B–D**: inferiorgrain) of two varieties (XC 6 and XC 31) at normal temperature (CK), normal temperature plus spermine (CK + Spm), normal temperature plus Spermidine (CK + Spd), high temperature (HT), high temperature plus spermine (HT + Spm), and high temperature plus Spermidine (HT + Spd) with the days after flowering in 2018 and 2019. Bars indicate SD (n = 3). The same letters within each panel imply no statistically significant differences(P < 0.05).

Under high temperature stress, the grain filling of XC 6and XC 31 could be significantly inhibited, (Fig. 1, Year, 2018). From 13 day after flowering (DAF) to 31DAF, the grain weight of two varieties in HT treatment was always significantly lower than that in CKtreatment. From the perspective of superior and inferiorfinal grain weight of the two varieties, the effect of HT treatment on inferior grain (reduced by 17% and 16%) was greater than that on superior grain (reduced by 11% and 16%), compared with CK, the quality of superior grain of XC 31 (reduced by 16%) decreased more than that of XC 6 (decreased by 11%), this indicates that there are differences in the tolerance of different varieties to high temperature stress, XC 6 is better than XC 31, and maintaining the grain filling ability of superior grains under high temperature stress is the main reason for the variety difference;From the 19 DAF, the quality of superior and inferior grains of two varieties under HT + Spm and HT + Spd was significantly higher than that under HT, but significantly lower than that under CK, indicating that exogenous Spm and Spd significantly alleviated the inhibiting effect of high temperature stress on wheat grain filling. Moreover, from the final weightof superior and inferiorgrains of the two varieties, Spm had a better alleviating effect than Spd. From the curve of grain filling rate, it can be seen that, except the HT treatment of XC 6, the grain filling of superior and inferior grains of other treatments basically followed the grain filling process showing a single peak change trend of increasing at first (the peak at about 13 DAF) and then decreasing.

The maximum grain filling rate, final grain weight and average grain filling rate of the two varieties under HT treatment were significantly lower than CK (Table 2). The active grain filling period of the two varieties was as follows: HT + Spd≥HT + Spm≥HT > CK(Table 2, Year, 2018), but the difference was not significant, showing that high temperature stress of two varieties at the early stage of grain-filling may have the effect of heat training andprolong the grain-filling period, and high temperature stress reduced grain weight mainly by reducing grain filling rate. The maximum grain filling rate, final grain weight, average grain filling rate and active grain filling period of the two varieties under HT + Spm and HT + Spd were higher than HT, indicating that exogenous spraying Spm and Spdcould alleviate the heat injury of grain filling, the wheat yield under high temperature stress can be increased.

**Table 2:** Grain-filling characteristics of two varieties (XC 6 and XC 31) at normal temperature (CK), normal temperature plus spermine (CK + Spm), normal temperature plus Spermidine (CK + Spd), high temperature (HT), high temperature plus spermine (HT + Spm), and high temperature plus Spermidine (HT + Spd) with the days after flowering in 2018and 2019.

Year	Varieties	Treatment	Final grain mass (g)		Maximumgrain filling rate (g·d^−1^)		Mean grain filling rate (g·d^−1^)		Grain filling period(d)
			Superior	Inferior	Superior	Inferior	Superior	Inferior	
2018	XC 6	CK	0.054^a^	0.042^a^	0.0051^a^	0.0040^a^	0.00169^a^	0.00131^a^	32^a^
		HT	0.048^d^	0.035^d^	0.0032^d^	0.0022^c^	0.00145^c^	0.00106^c^	33^a^
		HT + Spm	0.052^bc^	0.040^bc^	0.0035^bc^	0.0025^b^	0.00158^b^	0.00121^b^	33^a^
		HT + Spd	0.050^c^	0.038^c^	0.0037^b^	0.0027^b^	0.00147^c^	0.00112^c^	34^a^
	XC 31	CK	0.056^a^	0.042^a^	0.0037^a^	0.0031^a^	0.00170^a^	0.00127^a^	33^a^
		HT	0.047^d^	0.035^d^	0.0032^b^	0.0019^c^	0.00138^c^	0.00103^c^	34^a^
		HT + Spm	0.053^bc^	0.040^b^	0.0031^b^	0.0027^b^	0.00151^b^	0.00114^b^	35^a^
		HT + Spd	0.051^c^	0.038^c^	0.0037^a^	0.0029^ab^	0.00146^c^	0.00109^c^	35^a^
2019	XC 6	CK	0.0553^a^	0.0437^b^	0.0050^a^	0.0037^b^	0.00179^a^	0.00141^b^	31^a^
		HT	0.0507^c^	0.0377^d^	0.0033^d^	0.0023^e^	0.00169^d^	0.00126^d^	30^a^
		CK + Spm	0.0557^a^	0.0463^a^	0.0052^a^	0.0043^a^	0.00174^b^	0.00145^a^	32^a^
		CK + Spd	0.0547^a^	0.0453^a^	0.0053^a^	0.0041^a^	0.00171^bc^	0.00142^ab^	32^a^
		HT + Spm	0.0524^b^	0.0403^c^	0.0039^c^	0.0029^d^	0.00174^bc^	0.00134^c^	30^a^
		HT + Spd	0.0513^bc^	0.0393^c^	0.0043^b^	0.0034^c^	0.00171^bc^	0.00131^c^	30^a^
	XC 31	CK	0.0477^b^	0.0387^c^	0.0035^b^	0.0031^a^	0.00144^b^	0.00117^c^	33^a^
		HT	0.0423^e^	0.0327 ^f^	0.0025^d^	0.0020^c^	0.00125^e^	0.00096 ^f^	34^a^
		CK + Spm	0.0497^a^	0.0417^a^	0.0036^ab^	0.0033^a^	0.00151^a^	0.00126^a^	33^a^
		CK + Spd	0.0483^b^	0.0403^b^	0.0038^a^	0.0027^ab^	0.00146^b^	0.00122^b^	33^a^
		HT + Spm	0.0453^c^	0.0367^d^	0.0027^d^	0.0021^c^	0.00133^c^	0.00108^d^	34^a^
		HT + Spd	0.0437^d^	0.035^e^	0.0030^c^	0.0023^bc^	0.00128^d^	0.00103^e^	34^a^

Values (means, n = 3) followed by different letters among different treatments are significantly different according to the Duncan’s multiple range tests (P < 0.05).

### Polyamines in grains

**Figure 2:**
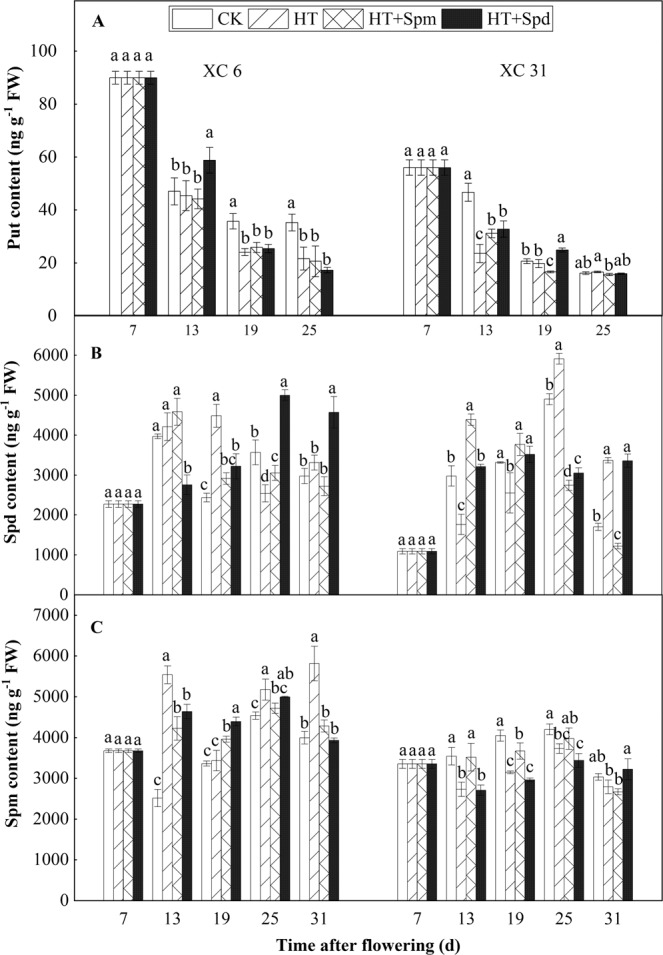
Changes in content of putrescine (**A**) PUT, spermidine (**B**) Spd and spermine (C) Spm of two varieties (XC 6 and XC 31) at normal temperature (CK), high temperature (HT), high temperature plus spermine (HT + Spm), and high temperature plus Spermidine (HT + Spd) with the days after flowering in 2018. Bars indicate SD (n = 3). The same letters within each panel imply no statistically significant differences.

With the grain filling process, the content of Put in the grains of the two varieties basically show a decreasing trend, while the content of Spmand Spdbasically show a trend of increasing first and then decreasing (Fig. 2). After HT treatment (13 DAF), the Put content in the grains of XC 6 decreased slightly compared with CK, the content of Spd was not significantly increased, while that of Spm was significantly increased; the content of Put, Spm, Spdin the grains of XC 31 were significantly decreased compared with CK, which indicated that there were differences in response to high temperature stress between different varieties. Compared with HT(13 DAF), HT + Spd significantly increased the content of Put in the grains of two varieties, both Spm and Spdcontent in the grains of XC 6 were significantly decreased, while in the grains of XC 31, Spdcontent was significantly increased andthe decrease of Spmcontent was not significant. Compared with HT, HT + Spmmade no significant difference in the Put and Spd content in the grains of XC 6, while the content of Spmwas significantly decreased, However, the contents of Put, Spm and Spd in XC 31 grains increased significantly. At 25 DAF, there was no significant difference in the content of Put in the grains of the two varieties among HT, HT + Spm and HT + Spd treatments, HT + Spm and HT + Spdmade the content of Spd in the grains of XC 6 significantly higher than HT, while of XC 31 significantly lower than HT. At 31 DAF, HT + Spdmade the Spdcontent in the grains of XC 6 significantly higher than that in the other three treatments(CK, HT and HT + Spm), and for XC 31 the Spm content in the grains wasthe highest among the four treatments (CK, HT, HT + Spm and HT + Spd).

### Antioxidant enzymatic activities in grain

**Figure 3:**
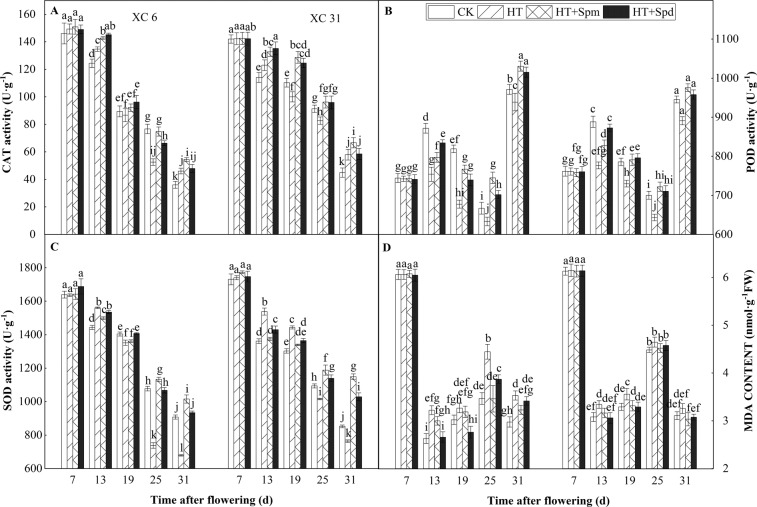
Changes in activity of catalase (**A**) CAT, peroxidase (**B**) POD, superoxide dismutase (**C**) SOD, and content of malonaldehyde (D) MDA of two varieties (XC 6 and XC 31) at normal temperature (CK), high temperature (HT), high temperature plus spermine (HT + Spm), and high temperature plus Spermidine (HT + Spd) with the days after flowering in 2018. Bars indicate SD (n = 3). The same letters within each panel imply no statistically significant differences.

With the grain filling process, the activities of CAT and SOD in the grains of the two varieties showed a decreasing trend(Fig. 3A–C), POD activity and MDA content showed a first increasing and then decreasing trend (Fig. 3B–D). After HT treatment (13 DAF), the activity of SOD and CAT and MDA content in the grains of the two varieties were significantly higher than CK, while POD activity of CK was significantly higher than that of HT, indicating that high temperature stress at the early stage of grainfilling led to destruction and lipid peroxidation of cell membrane, and XC 6 and XC 31 can quickly activate the antioxidant system to adapt to high temperature stress, mainly through increasing the activity of SOD and CAT. Compared with HT (13 DAF), HT + Spm and HT + Spd could increase the activity of CAT and POD and decrease SOD activity and MDA content of the two varieties, and the effect of Spd was better than that of Spm. At 25 DAF, the activity of SOD, POD and CAT in the grains of the two varieties under HT was significantly lower than those under CK; Compared with HT, HT + Spm and HT + Spd significantly increased the activity of SOD, POD and CAT, whileMDA content of grainswas decreasedsignificantly in XC 6 notsignificantly in XC 31, and the increasing or decreasingeffect of Spm was better than that of Spd, indicating that the damage of heat stress in the early stage of grainfilling is a continuous process, exogenous spraying polyamine to alleviate the damage on grain fillingcaused by high temperature stress is a dynamic process.

### Soluble sugar and proline content in grain

**Figure 4:**
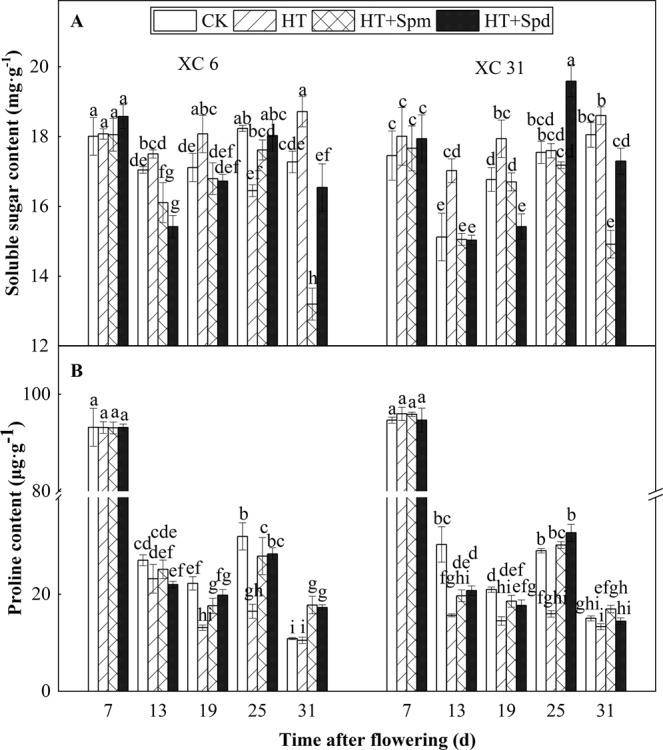
Changes in content of soluble sugar (A) SS and proline (B) Pro of two varieties (XC 6 and XC 31) at normal temperature (CK), high temperature (HT), high temperature plus spermine (HT + Spm), and high temperature plus Spermidine (HT + Spd) with the days after flowering in 2018. Bars indicate SD (n = 3). The same letters within each panel imply no statistically significant differences.

During the grain filling, the soluble sugar (SS) content in the grains of the two varieties under CK showed a change trend of first decreasing and then increasing, while under HT, it showed a wave change trend of first decreasing, then increasing and then decreasing (Fig. 4A). After HT (13 DAF), the contents of SS and proline (Pro) in the grains of XC 6 were not significantly different from those in CK, while for XC 31, they were significantly different, that is, compared with CK, SS increased by 13% and Pro decreased by 48%. Compared with CK, HT treatment made SS content in the grains of XC 6 significantly higher at 19 and 31 DAF and of XC 31 significantly higher at 13 and 19 DAF, indicatingthere were differences in the response of SS to high temperature stress between XC 6 and XC 31. After HT (13 DAF), Pro content in grains of the two varieties was significantly lower than CK at 19 and 25 DAF, while HT + Spm and HT + Spd could significantly increase the Pro content in the grains of two varieties at 25 DAFand significantly decrease the SS content at 13 and 19 DAF, indicating that exogenous spraying Spm and Spdunder high temperature stress had significant effects on the contents of SS and Pro in grains.

### Correlation analysis ofinvestigated parameters in the grains of wheat plants

The correlation analysis showed that grains weight (GW) was negatively correlated with the contents of PUT, CAT, MDA, Pro and activity of SOD in the grains, positively correlated with the contents of Spd and activity of POD, but not significantly correlated with the contents of Spm and SS (Table 3); The content of Put was negatively correlated with the content of Spd, and positively correlated with the contents of MDA, Pro and activity of SOD, CAT; The content of Spm was positively correlated with that of Spd; The content of Spd was negatively correlated with MDA and Pro contentand activity of SOD; SOD activity was positively correlated with CAT activity, MDA and Pro content, and negatively correlated with POD activity; POD activity was negatively correlated with CAT activity, MDA and SS content; CAT activity was significantly positively correlated with MDA and Pro content; MDA content was significantly positively correlated with SS and Pro content; SS content was positively correlated with Pro content.

**Table 3:** Correlation analysis of the investigated parameters in the grains of two varieties (XC 6 and XC 31) at normal temperature (CK), high temperature (HT), high temperature plus spermine (HT + Spm), and high temperature plus Spermidine (HT + Spd) with the days after flowering in 2018. Significant correlations at 0.05 levels were highlighted in bold.

	G W	Put	Spm	Spd	SOD	POD	CAT	MDA	S S	Pro
G W	1									
Put	−0.815**	1								
Spm	0.2	−0.053	1							
Spd	0.406**	−0.396*	0.321*	1						
SOD	−0.881**	0.710**	−0.295	−0.401*	1					
POD	0.320*	−0.187	−0.205	−0.12	−0.328*	1				
CAT	−0.885**	0.713**	−0.233	−0.24648	0.922**	−0.381*	1			
MDA	−0.639**	0.644**	−0.059	−0.450**	0.483**	−0.442**	0.471**	1		
S S	−0.116	0.18	0.08	−0.054	0.008	−0.450**	−0.013	0.461**	1	
Proline	−0.780**	0.828**	−0.142	−0.555**	0.695**	−0.304	0.645**	0.915**	0.343*	1

Investigated parameters: G W: grain weight; contents ofPut: putrescine; Spd: spermidine and Spm:spermine; MDA: malonaldehyde; S S: soluble sugar; Pro: proline; enzyme activities of SOD: superoxide dismutase; POD: peroxidase; CAT: catalase. ^*^Correlation is significant at the 0.05 level (2-tailed). ^**^Correlation is significant at the 0.01 level (2-tailed).

## Discussion

### Effects of PAs on the grain filling of wheat under heat stress

In our two-year experiment, high temperature stress treatment for 5 consecutive days at 7 days after flowering significantly decreased the yield of XC 6 and XC 31, which is caused by the decrease of GNP and grain weight (GWP and TGW). The decrease of grain weight is mainly related to the significant decrease of grain filling rate. This is consistent with the previous studies that wheat suffered high temperature stress during the grain filling period, resulting in the decrease of grain weight and filling rate^25,26^. It was also found that the inhibitory effect of high temperature stress on inferior grains was greater than that on superior grains, which was consistent with previous studies^11,27,28^. Previous studies have shown that rice (*Oryza sativa* L.)can cope with high temperature stress by accumulating polyamines under drought stress^29–31^. Exogenous application of polyamines increasedplant tolerance to drought or osmotic stress^32,33^, the contents of Spd and Spm were positively correlated with the grain weight of rice, and negatively correlated with the Put content^24^, while some studies also suggested that there was no significant correlation between the grain filling rate and Put content^34^. In this study, it was found that exogenous spraying Spm and Spd could significantly increase the final grain weight of superior and inferior grains in two varieties under high temperature stress. Correlation analysis indicated that the endogenous Spd contents of the grains were positively and very significantly correlated with grain weight, while endogenous put content was negatively and very significantly correlated with grain weight. Endogenous Spm content was positively correlated with grain weight, but not significantly, and significantly positively correlated with Spd content in grains. These results showed that exogenous Spd and Spm could effectively alleviate the injury of high temperature stress on the grain filling of XC 6 and XC 31, and this alleviating process is related to the change of endogenous polyamine content regulated by exogenous polyamine. In this study, it was not found that HT, HT + Spd and Spm had a significant effect on the grain filling time. This may be due to in shihezi, xinjiang, China, at the late stage of wheat grain filling (about June 30), daytime temperatures can reach as high as 37°C, grain filling between treatments is forced to stop, it is difficult to observe the difference in grain filling period between treatments in this case. So, we plan to conduct experiments in the greenhouse next, control the temperature at the later stage of grain filling and make a further observation.

### Relationship among antioxidant enzymatic activities, osmotic adjustable substance and grain fillingof wheat

High temperature is a serious abiotic stress factor, which affects various physiological and biochemical changes in plant growth and development^35,36^, Plants have developed many mechanisms to alleviate the damage effects of heat stress, ROS can induce the increase in the expression of protective enzymes, such as SOD, POD, CAT and other antioxidant enzymes, MDA is the final product of plasma membrane peroxidation, and its molality concentration can reflect the degree of plant damage^37–40^, In this study, it was found that in the early stage of high temperature stress, SOD activity, CAT activity and MDA content in the two varieties were significantly increased compared with CK, this is consistent with the research result that high temperature treatment in early stage could enhance SOD and CAT activities in wheat leaves^41^, POD activity was inhibited by high temperature, it is consistent with the research of Zhang *et al*. (2015) that POD activity was decreased by high temperature^42^. This study also found that SOD, POD and CAT activities in the grains of the two varieties were lower than CK in the later stage of grainfilling under HT, indicating that the damage of cell membrane caused by high temperature stress in the early stage of grain filling resulted in lipid peroxidation of cell membrane, Through increasing SOD and CAT activities, wheatcould cope with high temperature stress, however, the activities of SOD, CAT and POD decreased in the later grainfilling period, which led to the intensification of membrane lipid peroxidation. Previous studies have shown that PA, a necessary compound for many cell functions (including the response to environmental stress), is not only a direct protective substance, but also a signal to trigger some adaptive mechanisms^43,44^, and can scavenge active oxygen radicals, stabilize the structure of biological membrane, and interact with biological macromolecules^45,46^. In this study, it was found that under high temperature stress (13 DAF), exogenous Spm and Spd could significantly increase the activity of CAT and POD, and decrease the activity of SOD and MDA content inthe grains of the two varieties; At 25 DAF, exogenous Spm and Spd could significantly increase the activity of SOD, POD and CAT and decrease the content of MDA in the grains of the two varieties under high temperature stress, indicating that alleviating effect of exogenous spraying PAsonthe damage ofwheat grain fillingcaused by high temperature stress was a dynamic process. Further study is needed on the molecular mechanism of polyamines increasing the activity of antioxidant enzymes.

Many plants can sustain growth in abiotic conditions, one of the key adaptive mechanisms isaccumulation of amino acids, sugars, sucrose, and amines (such as polyamines) works as osmotic adjustor under abiotic stresses^47–49^. This study found that after HT (13 DAF), SS content in the grains of two varieties increased, which was consistent with the results of Gao *et al*. study^50^, however, the decrease of Pro content in grains was different from previous studies which showed that Pro content in leaves increased under high temperature stress^51^, this difference in the results may be due to the different responses of different tissues of wheat to high temperature stress;Compared with HT(13 DAF), SS content of XC 6 was not significantly increased, while that of XC 31 was significantly increased, indicating that the effect of heat stress on XC 31 was greater than XC 6; Exogenous Spm and Spd could significantly increase the content of Pro (19 and 25 DAF), and significantly decrease the content of SS (13 and 19 DAF) in grains of two varieties under HT, indicating that exogenous spraying Spm and Spdhad significant effectson the content of SS and Pro in the grains under high temperature stress.

## Conclusions

Our results showed that high temperature stress significantly inhibited grain filling of XC 6 and XC 31, and XC 6 and XC 31 have different responses to high temperature stress in two years. Exogenously spraying Spd and Spm could alleviate the inhibition of grain filling under high temperature stress. Alleviating process is closely related to endogenous polyamine content (Put, Spd and Spm), antioxidant enzyme activity (SOD, POD CAT) and osmotic adjustment substances content (SS and Pro) in grains. Whether exogenous polyamines can alleviate high temperature stress through modulating gene expression needs further study.

## Materials and methods

### Study site description

This study was conducted from 2018-2019 at a research station of Shihezi University, Xinjiang, in northwestern China (45°19′N, 74°56′E). The annual mean precipitation of the experimental station is 550 mm, the average annual maximum and minimum temperatures during the crops growing season were 36.9 °C and 9.4 °C, respectively, and the annual mean temperature is 12.9 °C. The total yearly sunshine duration is 2196 h, and the period is 220 days. The soil at the experiment site is moderate fertility, the readily available N, P and K quantities were 0.058 g kg^−1^, 0.025 g kg^−1^, and 0.149 g kg^−1^ respectively, The organic matter concentration of the 0–20 cm topsoil was 12.34 g kg^−1^, and the pH was 7.35 (2018)^52^.

## Experimental design and treatments

### The first experiment

The experiment was performed in the field. Two wheat varieties^53^, XC 6 (a heat-resistant variety) and XC 31 (a heat-sensitive variety), were grown. The seeds were sown on 24 March in 2018. The sowing density was 150 kg hm^−2^, with a row spacing of 0.20 m. The diammonium phosphate (N content was 16.5%, containing P_2_O_5_ 47.5%) 155 kg hm^−2^ were used as base fertilizer; 70, 150, 80 and 80 kg hm^–2^urea were applied respectively in the 3-leaf stage, jointing stage, booting stage and filling stage (drip fertilization).

The experiment was a 2 × 2 × 2 (two levels of temperature and two polyamines and two varieties) factorial design, with 8 treatment combinations. Each of the treatments contained three plots as replicates in a complete randomized block design. Before the high temperature treatment, the plants with the same growth (Same flowering time) were selected and marked. At 7 day after flowering (DAF), the shed was kept in the field for 5 days, and the plastic film was put down at 10:00–18:00 h each day (20 cm off the ground for ventilation) to increase the temperature. The temperature inside and outside the shed during the treatment was recorded with an automatic thermometer (the thermometer was suspended 15 cm above the wheat canopy). The temperature changes during the treatment period in 2018 and 2019 are shown in Table 4. It can be seen from the average temperature inside and outside the shed that the effect of high temperature stress has been achieved (Table 4).

**Table 4:** Changes of temperature inside and outside the shed during the treatment periods in 2018 and 2019.

Years	Date (m-d)	Temperature(°C)					
		Outside the shed			Inside the shed		
		Lowest	Highest	Average	Lowest	Highest	Average
2018	06–07	27.8	34.4	31.1	26.7	42.4	34.88
	06–08	28.1	35.1	30.45	28.2	37.9	32.29
	06–09	27	36.5	32.33	29.5	38.9	34.62
	06–10	27.7	35.8	32.08	27.7	41.7	37.75
	06–11	26.4	36.1	30.93	28	41.7	34.24
2019	06–05	23.7	34.8	29.69	23.9	41.6	34.24
	06–06	29.4	35.4	31.94	29.5	41.6	37.09
	06–07	18.2	30.9	27.02	18.3	40.4	32.78
	06–08	29.7	33.3	31.8	28.9	37.9	34.47
	06–10	24.4	30.5	26.77	29.5	39.4	32.14

The temperature was not recorded on June 9, 2019 because of rain, so it was postponed for one day.

Treatment and labeling methods are: control (normal growth conditions outside the greenhouse), recorded as CK; artificially simulated high temperature, recorded as HT; artificially simulated high temperature plus exogenous application of spermine (1 mM L^−1^), recorded as HT + Spm; artificially simulated high temperature plus spermidine (1 mM L^−1^) was recorded as HT + Spd. Spm and Spd were purchased from Sigma Company (USA) with purity Spd were purchased froely. Exogenous spraying PAs started from the day before high temperature treatment, lasting for 5 days, at 20:00 h of everyday, PAs were sprayed on the flag leaves and panicles, each for 20 ml (CK and HT spraying with water).

### The second experiment

The experiment was also performed in the field. The same two varieties, XC 6 and XC 31, were used in exogenous PAs application treatments. Each variety received six treatments at 7 day after flowering (DAF), as follows: (1) CK: normal growth conditions outside the greenhouse; (2) CK + Spm: normal growth conditions outside the greenhouse plus exogenous application of spermine (1 mM L^−1^); (3) CK + Spd: normal growth conditions outside the greenhouse plus spermidine (1 mM L^−1^); (4) HT: artificially simulated high temperature; (5) HT + Spm: artificially simulated high temperature plus exogenous application of spermine (1 mM L^−1^); (6) HT + Spd: artificially simulated high temperature plus spermidine (1 mM L^−1^).

At 7 DAF, 1 mM L^–1^ Spm and 1 mM L^–1^Spd were sprayed on the flag leaves and panicles with a sprayer. Exogenous spraying PAs started from the day before high temperature treatment, lasting for 5 days, at 20:00 h of everyday. All of the solutions contained 0.1% (V/V) ethanol and 0.01% (V/V) Tween −20. The same volume of deionized water containing the same concentrations of ethanol and Tween −20 was applied to CK and HT. Each treatment had three replicates with a completely randomized block design. The Spm and Spd were purchased from Sigma Company (USA).

### Measurement

Two hundred panicles that flowered on the same day were chosen and tagged in each plot. Tagged spikes from each plot were sampled at 5-d intervals from 7 DAF to maturity. All grains from each spike were removed. Grains on a spike were divided into superior grains and inferior grains. The most basal grains in the middle panicles (4 to 12 spikelets) from the bottom of a spike were considered superior grains, and the most distal grains in the middle panicles (4 to 12 spikelets) from the bottom of a spike were considered inferior grains^54^. Half of the sampled grains were used for measurements of PAs, antioxidant enzymatic activities (SOD, POD and CAT) and content of osmotic adjustment substances (SS and Pro) and MDA. The other half of the grains were dried at 70 °Cand weighed until a constant weight was observed.

### Yield and yield components

Randomly selected 15 plants of wheat, to determine the grain number per panicle (GNP), grain weight per panicle (GWP); randomly selected 1000 grains of each treatment to test the thousand grain weight (TGW), repeating for 3 times to measure TGW; Theoretical yield (TY) = number of panicle per hectare (300×10000) × grain number per panicle (GNP) × thousand grain weight (TKW) × 10^–6^ × 85%.

### Grainfilling process

Samples were taken at 0, 5, 15, 20 and 25 days of high temperature treatment, and wheat grains were divided into superior and inferior grains according to the classification method. After fixingfor 30 minutesat 105 °C, they were dried and weighed at 70 °C. The grain-filling period=The date of death of wheat plants (more than 50%)–The date of flowering of wheat plants(more than 50%).

### Detection of free and soluble-conjugated PAs

Samples were taken at 0, 5, 15, 20 and 25 days of high temperature treatment. Spd, Spm, and Put were extracted and measured according to Cheng *et al*.^55^. Concentrations of free and conjugated PAs were determined using a modified high performance liquid chromatography (HPLC) method. Briefly, approximately 0.5 g fresh weight (FW) of samples was homogenized in a pre-chilled mortar and pestle in 5 mL 10% perchloric acid, incubated on ice for 1.5 h, and then centrifuged at 18514 g for 20 min at 4 °C. Seven micro liter benzoyl chloride and 1 mL 2 M NaOH were then added to 500 μL of the supernatant. The reactions were allowed to proceed at 37 °C for 30 min and then 2 mL ether and 2 mL saturated NaCl were added to the reactions. The reactions were shaken for 5 min and then 1 mL of the ether phase was removed and dried under vacuum. The dried reactions were re-dissolved in 100 μL methanol before HPLC analysis. The HPLC was performed on an Agilent 1200 system (Agilent, USA) with an Agilent XDB-C18 (4.6 mm × 150 mm) column. The HPLC conditions were as follows: liquid phase with a methanol: water ratio of 60:40 (v/v), 1 mL/min of flow rate, 10 μL of sample per injection, detection at 30 °C with a wave length of 254 nm, and 30 min of retention time. Peak areas and retention times were measured by comparison with standard Put, Spd, and Spm. The concentrations of PAs (ng of PAs g^−1^ fresh callus weight) were determined using a standard curve prepared with known amounts of standard Put, Spd, and Spm. The assays were technically repeated three times.

### Determination of antioxidant enzymes activity

Samples were taken at 0, 5, 15, 20 and 25 days of high temperature treatment. Generally, 0.1 g grain sample wasground by adding 0.9 mL extracting solution of SOD, POD and CAT, respectively, at 0 °C using test kit(Jiancheng, Nanjing, China)^8^. The samples were centrifugedat 3500 r/min for 10 min. For the SOD activity, the 0.02 mLsupernatant was added with 0.02 mL reagent I and 0.2 mLreagent II and then kept for 20 min at 37 °C. For the PODactivity, 2.4 mL reagent I, 0.3 mL reagent II and 0.2 mLreagent III were mixed with 0.1 mL sample. The mixturewas kept for 30 min at 37 °C and then 1.0 mL reagent IVwas added. The solution was centrifuged at 3500 r/min lasting10 min. The activity of SOD and POD were determinedat 450 nm and 420 nm using a UV-2450 spectrophotometer(Shimadzu, Japan), respectively. For the CAT activity, the 0.02 mL sample was mixed with3 mL substrate reaction solution. The absorbance at 240 nmwas measured immediately and 60 s later using the UV spectrophotometer.

### Detection of MDA, soluble sugar, and proline content and proline content

Samples were taken at 0, 5, 15, 20 and 25 days of high temperature treatment. The 0.2 g grain sample was ground by adding1.8 mL extracting solution using MDA, SS and Pro testkit (Jiancheng, Nanjing, China)^8^. The samples were centrifugedat 3,500 r/min lasting 10 min. Afterwards, 0.05 mLsupernatant was mixed with 0.1 mL reaction solution, wellblended and water-bathed for 20 min at 95 °C to determineMDA content. Meanwhile, 0.5 mL sample was mixed with1 mL reagent I and 1 mL reagent II and the mixture waswater-bathed for 30 min at 95 °C to detect proline content. Then, the MDA, SS, and Pro content were measured at 532 nm, 620 nm and 520 nm, respectively, using a UV-2450 spectrophotometer(Shimadzu, Japan).

### Statistical analysis

Three independent repetitions were performed for each experiment, and representativedata are presented. The results were the means of at least 3 replicates for measurements of the grain weight, yield trait, spectrophotometric and HPLC determinations. The data were statistically evaluated using the standarddeviation and Duncan’s multiple range tests methods. The SPSS 22.0 statistical program (Statistical Package for the Social Sciences)was used to examine correlations between the parameters. The PAs, antioxidant enzyme activity (SOD, POD and CAT) and the content of osmotic adjustable substances (SS, and Pro) and MDA, were presented as the date in 2018–2019.

## Supplementary information

Dataset 1.

## Data Availability

All data generated or analysed during this study are included in this published article.

## References

[1] Porter JR, Gawith M (1999). Temperatures and the growth and development of wheat: a review. European J.of Agron..

[2] Asseng S, Foster I, Turner NC (2011). The impact of temperature variability on wheat yields. Global Change Biol..

[3] Fink AH (2010). The 2003 European summer heatwaves and drought? Synoptic diagnosis and impacts. Weather..

[4] Gill SS, Tuteja N (2010). Reactive oxygen species and antioxidant machinery in abiotic stress tolerance in crop plants. Plant Physiol. Biochem..

[5] Pál M, Szalai G, Janda T (2015). Speculation: Polyamines are important in abiotic stress signaling. Plant Sci..

[6] Hayat S (2012). Role of proline under changing environments: a review. Plant Signal &Behav..

[7] Zhang Q, Song X, Bartels D (2018). Sugar metabolism in the desiccation tolerant grass, Oropetium thomaeum, in response to environmental stresses. Plant Sci..

[8] Zhou, R. *et al*. Oxidative damage and antioxidant mechanism in tomatoes responding to drought and heat stress. *Acta Physiol. Plantarum*. **41** (2019).

[9] Sarafraz-Ardakani MR, Khavari-Nejad RA, Moradi F, Najafi F (2014). Abscisic acid and cytokinin-induced osmotic and antioxidant regulation in two drought-tolerant and drought-sensitive cultivars of wheat during grain filling under water deficit in field conditions. Notulae Sci. Biologicae..

[10] Janda T, Khalil R, Tajti J, Pál M, Darkó Éva (2019). Responses of young wheat plants to moderate heat stress. Acta Physiol. Plantarum..

[11] Wollenweber B, Porter JR, Schellberg J (2010). Lack of interaction between extreme high-temperature events at vegetative and reproductive growth stages in wheat. J. of Agron.& Crop Sci..

[12] Ortiz R (2008). Climate change: Can wheat beat the heat?. Agriculture Ecosystems & Env..

[13] Ainsworth EA, Ort DR (2010). How do we improve crop production in a warming world?. J. Plant Physiol..

[14] Wahid A, Gelani S, Ashraf M, Foolad MR (2007). Heat tolerance in plants: An overview. Env.& Exp. Bot..

[15] Alcázar R (2010). Putrescine accumulation confers drought tolerance in transgenic Arabidopsis plants over-expressing the homologous Arginine decarboxylase 2 gene. Plant Physiol. Biochem..

[16] Bais HP, Ravishankar GA (2003). Synergistic effect of auxins and polyamines in hairy roots ofCichorium intybusL. during growth, coumarin production and morphogenesis. Acta Physiol. Plantarum..

[17] Hussain SS, Ali M, Ahmad M, Siddique KHM (2011). Polyamines: Natural and engineered abiotic and biotic stress tolerance in plants. Biotechnology Adv..

[18] Shi HT, Chan ZL (2014). Improvement of plant abiotic stress tolerance through modulation of the polyamine pathway. J. of Integrative Plant Biology..

[19] Zhang, C. M., Zou, Z. R., Zhang, Z. X. & Huang, Z. Effects of exogenous spermidine on photosynthesis of different tomato seedlings under drought stress. *Agr. Res. Arid Areas*. **28**, 182–187. [In Chinese] (2010).

[20] Besford RT, Richardson CM, Campos JL, Tiburcio AF (1993). Effect of polyamines on stabilization of molecular complexes in thylakoid membranes of osmotically stressed oat leaves. Planta..

[21] Song, Y. J., Diao, Q. N. & Qi, H. Y. Advances in research on polyamine metabolism and plant stress resistance. *Chin.Vegetab*. **1**, 36–42 [In Chinese] (2012).

[22] Liang YL, Lur HS (2002). Conjugated and Free Polyamine Levels in Normal and Aborting Maize Kernels. Crop Sci..

[23] Yang J, Cao YY, Zhang H, Liu LJ, Zhang JH (2008). Involvement of polyamines in the post-anthesis development of inferior and superior spikelets in rice. Planta.

[24] Tan, G. L. *et al*. Post-anthesis changes in concentrations of polyamines in superior and inferior spikelets and their relation with grain filling of super rice. *Acta Agron. Sin*. **35**, 2225–2233 [in Chinese] (2009).

[25] Mao H (2018). Genetic analysis of heading date in winter and spring wheat. Euphytica..

[26] Battisti DS, Naylor RL (2009). Historical warnings of future food insecurity with unprecedented seasonal heat. Science..

[27] Zhao H, Dai T, Jing Q, Jiang D, Cao WX (2007). Leaf senescence and grain filling affected by post-anthesis high temperatures in two different wheat cultivars. Plant Growth Regulation..

[28] Shah NH, Paulsen GM (2003). Interaction of drought and high temperature on photosynthesis and grain-filling of wheat. Plant & Soil..

[29] Liu Y (2016). Effect of polyamines on the grain filling of wheat under drought stress. Plant Physiol.& Biochem..

[30] Capell T, Bassie L, Christou P (2004). Modulation of the polyamine biosynthetic pathway in transgenic rice confers tolerance to drought stress. Proc. Natl. Acad. Sci. USA.

[31] Yang J, Zhang J, Liu K, Wang Z, Liu L (2007). Involvement of polyamines in the drought resistance of rice. J. Exp. Bot..

[32] Shi H, Ye T, Chan Z (2013). Comparative Proteomic and Physiological Analyses Reveal the Protective Effect of Exogenous Polyamines in the Bermudagrass (*Cynodon dactylon*) Response to Salt and Drought Stresses. J. Proteome Res..

[33] Shi J (2010). Spermine pretreatment confers dehydration tolerance of citrus *in vitro* plants via modulation of antioxidative capacity and stomatal response. Tree Physiol..

[34] Liu Y, Gu DD, Wu W, Wen XX, Liao YC (2013). The Relationship between Polyamines and Hormones in the Regulation of Wheat Grain Filling. Plos One..

[35] Shah F (2011). Impact of high-temperature stress on rice plant and its traits related to tolerance. J. of Agricultural Sci..

[36] Liu XZ, Huang BR (2000). Heat Stress Injury in Relation to Membrane Lipid Peroxidation in Creeping Bentgrass. Crop Sci..

[37] Zandalinas SI, Rivero RM, Martínez V, Gómez-Cadenas A, Arbona V (2016). Tolerance of citrus plants to the combination of high temperatures and drought is associated to the increase in transpiration modulated by a reduction in abscisic acid levels. Bmc Plant Biology..

[38] Xu S, Li JL, Zhang XQ, Wei H, Cui LJ (2006). Effects of heat acclimation pretreatment on changes of membrane lipid peroxidation, antioxidant metabolites, and ultrastructure of chloroplasts in two cool-season turfgrass species under heat stress. Env.& Exp. Bot..

[39] Park S (2013). Melatonin-rich transgenic rice plants exhibit resistance to herbicide-induced oxidative stress. J. of Pineal Research..

[40] Mittler R (2002). Oxidative stress, antioxidants and stress tolerance. Trends in Plant Sci..

[41] Jiang, C. M., Yin,Y. P., Liu, X. & Wang, Z. L. Response of flag leaf lipid peroxidation and protective enzyme activity of wheat cultivars with different heat tolerance to high temperature stress after anthesis. *Acta Agron. Sin* **1**, 143–148 [In Chinese] (2007).

[42] Zhang, Y. H. *et al*. Effect of high temperature on photosynthetic capability and antioxidant enzyme activity of flag leaf and non-leaf organs in wheat. *Acta Agron. Sin*. **41**, 136–144 [In Chinese] (2015).

[43] Szalai G (2017). Comparative analysis of polyamine metabolism in wheat and maize plants. Plant Physiol Biochem..

[44] Pál M (2018). Interaction of polyamines, abscisic acid and proline under osmotic stress in the leaves of wheat plants. Sci. Rep..

[45] Drolet G, Dumbroff EB, Legge RL, Thompsona JE (1986). Radical scavenging properties of polyamines. Phytochemistry (Oxford)..

[46] Roberts DR, Thompson EBDE (1986). Exogenous polyamines alter membrane fluidity in bean leaves –a basis for potential misinterpretation of their true physiological role. Planta..

[47] Seki M, Umezawa T, Urano K, Shinozaki K (2007). Regulatory metabolic networks in drought stress responses. Current Opinion in Plant Biology..

[48] Yordanov I, Velikova V, Tsonev T (2000). Plant responses to drought, acclimation, and stress tolerance. Photosynthetica..

[49] Liu C (2011). Effect of drought on pigments, osmotic adjustment and antioxidant enzymes in six woody plant species in karst habitats of southwestern China. Env.& Exp. Bot..

[50] Gao, H. Y. *et al*. Physiological response of rice resistance to high temperature drought stress during grout and fruiting period. *Ecological env. monitoring of three gorges*. **2**, 11–27 [In Chinese] (2017).

[51] Jin, L. Z. *et al*. Effects of high temperature stress on physiological and biochemical characteristics of different tolerant soybean varieties. *Soybean sci*. **38**, 63–71 [In Chinese] (2019).

[52] Jing JG, Guo SY, Li YF, Li WH (2019). Effects of polyamines on agronomic traits and photosynthetic physiology of wheat under high temperature stress. Photosynthetica..

[53] Li, Z. F. *et al*. Evaluation of heat tolerance of spring wheat varieties in Xinjiang. *Triticeae Crop*. **11**, 1497–1502 [In Chinese] (2017).

[54] Jiang D, Cao WX, Dai TB, Jing Q (2003). Activities of key enzymes for starch synthesis in relation to growth of superior and inferior grains on winter wheat (*Triticum aestivum* L.) spike. Plant Growth Regulation..

[55] Cheng WH (2015). Polyamine and Its Metabolite H_2_O_2_ Play a Key Role in the Conversion of Embryogenic Callus into Somatic Embryos in Upland Cotton (*Gossypium hirsutum*L.). Frontiers in Plant Sci..

