# Supplementary material for: The alleviating effect of exogenous polyamines on heat stress susceptibility of different heat resistant wheat (Triticum aestivum L.) varieties
Source: Sci Rep. 2020 May 4;10:7467. doi: 10.1038/s41598-020-64468-5 (PMC7198572; doi:10.1038/s41598-020-64468-5)
Supplement: Supplementary file 1 — Dataset 1. [file 41598_2020_64468_MOESM1_ESM.pdf]

The alleviating effect of exogenous polyamines on heat stress susceptibility of different heat resistant wheat (*Triticum aestivum* L.) varieties

Jianguo Jing, Suyan Guo, Youfang Li, and Weihua Li*

2018

	Grains numbers per panicle	Weight per panicle	Thousand grains weight
XC6HT+Spm1	43	1.97	42.39
	49	2.07	
	40	2.12	
	40	2.02	
	44	2.02	
	43.2	2.04	
XC6HT+Spm2	49	2.01	42.47
	32	1.97	
	44	2.09	
	40	2.12	
	52	2.03	
	43.4	2.044	
XC6HT+Spm3	47	1.75	42.68
	48	2.07	
	41	2.83	
	37	1.64	
	44	1.95	
	43.4	2.048	
XC6HT+Spd1	45	1.91	41.89
	35	1.41	
	42	1.71	
	35	1.61	
	37	2.33	
	38.8	1.794	
	33	1.65	41.41
	45	1.76	
	49	1.62	

	39	2.76	
	29	1.25	
XC6HT+Spd2			
	39	1.808	
	37	1.55	42.14
	41	1.57	
	39	1.29	
	34	2.52	
	45	1.99	
XC6HT+Spd3			
	39.2	1.784	
	34	1.51	40.09
	33	2.31	
	47	1.32	
	35	1.23	
	37	2.32	
XC6HT1			
	37.2	1.738	
	37	1.58	39.83
	35	1.86	
	43	1.63	
	31	1.83	
	39	1.64	
XC6HT2			
	37	1.708	
	33	1.05	39.51
	37	1.26	
	38	1.87	
	38	2.34	
	38	2.02	
XC6HT3			
	36.8	1.708	
	47	2.33	46.15

	49	2.42	
	42	2.14	
	43	2.11	
XC6CK1	47	2.66	
	45.6	2.332	
	45	2.51	45.83
	42	2.66	
	50	1.95	
	47	2.28	
XC6CK2	40	1.99	
	44.8	2.278	
	43	2.51	45.79
	46	2.51	
	45	2.68	
	51	1.73	
XC6CK3	39	1.89	
	44.8	2.264	
	41	1.86	43.06
	46	1.73	
	45	1.86	
	45	1.97	
XC31HT+Spm1	44	1.75	
	44.2	1.834	
	45	2.11	42.96
	40	2.24	
	46	1.04	
	45	2.01	
XC31HT+Spm2	44	1.62	
	44	1.804	

	53	2.27	43.21
	50	2.12	
	47	1.66	
	37	1.62	
XC31HT+Spm3	37	1.65	
	44.8	1.864	
	50	2.49	43.59
	38	1.31	
	35	1.44	
	49	2.21	
XC31HT+Spd1	38	1.91	
	42	1.872	
	40	2.14	43.49
	40	2.47	
	42	1.54	
	44	1.48	
X31HT+Spd2	44	1.69	
	42	1.864	
	36	1.38	44.22
	45	1.95	
	44	2.54	
	41	2.03	
XC31HT+Spd3	45	1.41	
	42.2	1.862	
	27	0.84	39.12
	34	1.97	
	44	1.91	
	36	1.54	
XC31HT1	36	0.73	
	35.4	1.398	

	31	1.03	38.88
	44	1.32	
	30	1.29	
	43	1.74	
XC31HT2	32	1.61	
	36	1.398	
	35	1.25	39.55
	40	1.36	
	44	1.06	
	32	1.32	
XC31HT3	30	1.97	
	36.2	1.392	
	53	2.68	45.85
	46	1.21	
	44	1.88	
	41	2.22	
XC31CK1	56	1.76	
	48	1.95	
	54	1.51	46.32
	46	2.35	
	47	2.21	
	46	2.08	
XC31CK2	49	2.29	
	48.4	2.088	
	55	2.02	45.94
	57	1.39	
	45	2.53	
	39	2.16	
XC31CK3	47	2.45	
	48.6	2.11	

	2019		
	Grains numbers per panicle	Weight per panicle	Thousand grains weight
XC6CK1	41	2.14	50.88
	49	2.37	
	51	2.27	
	49	2.1	
	41	2.9	
	46.2	2.356	
XC6CK2	47	2.22	49.8
	52	2.11	
	38	2.17	
	51	2.14	
	50	3.27	
	47.6	2.382	
XC6CK3	45	2.11	50.23
	51	2.61	
	46	2.24	
	51	2.65	
	44	2.27	
	47.4	2.376	
XC6HT1	36	1.62	45.24
	39	1.77	
	40	2.02	
	39	2.06	
	46	1.91	
	40	1.876	

	29	1.7	45.28
	36	1.77	
	44	2.33	
	44	2.26	
XC6HT2	41	1.43	
	38.8	1.898	
	37	2.01	45
	45	2.53	
	40	1.07	
	43	2.35	
XC6HT3	31	1.58	
	39.2	1.908	
	48	2.56	52.76
	45	2.36	
	48	2.39	
	48	2.56	
XC6Spm1	46	2.68	
	47	2.51	
	48	2.55	53.07
	53	2.56	
	45	2.53	
	45	2.46	
XC6Spm2	50	2.57	
	48.2	2.534	
	42	1.78	51.47
	54	2.87	
	51	2.44	
	51	2.78	
XC6Spm3	45	2.71	
	48.6	2.516	

	49	2.49	50.82
	44	2.39	
	48	2.52	
	49	2.63	
XC6Spd1	49	2.3	
	47.8	2.466	
	51	2.06	50.63
	44	2.81	
	43	2.45	
	53	2.19	
XC6Spd2	47	2.74	
	47.6	2.45	
	45	2.66	50.34
	50	2.31	
	44	2.36	
	51	2.37	
XC6Spd3	47	2.55	
	47.4	2.45	
	41	2.25	49.11
	40	1.93	
	48	2.43	
	36	2.24	
XC6HT+Spm1	42	2.23	
	41.4	2.216	
	38	1.99	48.87
	46	2.21	
	39	2.31	
	37	2.27	
XC6HT+Spm2	46	2.29	
	41.2	2.214	

	31	1.61	48.54
	39	2.1	
	51	2.81	
	46	2.37	
XC6HT+Spm3	47	2.54	
	42.8	2.286	
	44	1.91	48.39
	47	2.47	
	40	2.18	
	47	2.22	
XC6HT+Spd1	34	1.89	
	42.4	2.134	
	40	1.94	48.25
	41	2.18	
	41	2.2	
	36	2.25	
XC6HT+Spd2	49	2.2	
	41.4	2.154	
	48	2.17	48.29
	41	2.34	
	43	2.01	
	35	2.2	
XC6HT+Spd3	40	2.16	
	41.4	2.176	
	43	1.95	45.81
	36	1.57	
	41	2.41	
	54	2.52	
XC31CK1	39	1.73	
	42.6	2.036	

	42	2.29	45.94
	46	2	
	47	1.94	
	32	1.93	
XC31CK2	49	1.91	
	43.2	2.014	
	46	2.19	45.65
	43	1.62	
	39	2.22	
	43	2.29	
XC31CK3	46	2.11	
	43.4	2.086	
	40	1.55	43.29
	40	1.44	
	42	1.28	
	33	1.49	
XC31HT1	38	1.45	
	38.6	1.442	
	37	1.72	42.72
	35	1.5	
	46	1.37	
	38	1.11	
XC31HT2	36	1.53	
	38.4	1.446	
	43	1.27	42.89
	38	1.25	
	40	1.46	
	29	1.41	
XC31HT3	44	1.79	
	38.8	1.436	

	50	2.56	45.89
	45	1.92	
	49	2.22	
	44	2.41	
	42	2.15	
XC31Spm1			
	46	2.252	
	45	2.25	46.46
	43	2.06	
	44	2.73	
	52	1.97	
	46	2.03	
XC31Spm2			
	46	2.208	
	48	2.1	45.99
	47	2.15	
	44	2.3	
	48	2.06	
	41	2.5	
XC31Spm3			
	45.6	2.222	
	37	2.5	46.59
	42	2.18	
	50	2.81	
	48	2.03	
	41	1.76	
XC31Spd1			
	43.6	2.256	
	43	1.96	45.21
	42	2.54	
	46	1.79	
	45	2.25	
	43	2.32	
XC31Spd2			

	43.8	2.172	
	47	2.35	45.86
	41	2.14	
	42	2.04	
	49	1.91	
	40	1.96	
XC31Spd3			
	43.8	2.08	
	44	1.66	44.1
	47	1.84	
	42	2.08	
	39	1.92	
	38	1.61	
XC31HT+Spm1			
	42	1.822	
	48	1.78	43.09
	46	1.72	
	39	1.97	
	38	1.78	
	35	1.84	
XC31HT+Spm2			
	41.2	1.818	
	46	1.75	44.5
	47	1.91	
	42	2.02	
	34	1.65	
	41	1.74	
XC31HT+Spm3			
	42	1.814	
	43	1.58	43.27
	49	1.99	
	40	1.91	
	38	1.78	
	40	1.95	
XC31HT+Spd1			

XC31HT+Spd1			
	42	1.842	
	41	1.25	43.91
	43	2.1	
	44	1.92	
	40	1.74	
XC31HT+Spd2	38	1.66	
	41.2	1.734	
	42	1.82	43.44
	35	1.59	
	45	1.67	
	44	1.68	
XC31HT+Spd3	43	1.44	
	41.8	1.64	

2018

Varieties	Treatment	Grains numbers per panicle	STDEV		
XC6	HT+Spm1	43.2	0.115470054	43.2	43.333333
XC6	HT+Spm2	43.4		43.4	
XC6	HT+Spm3	43.4		43.4	
XC6	HT+Spd1	38.8	0.2	38.8	39
XC6	HT+Spd2	39		39	
XC6	HT+Spd3	39.2		39.2	
XC6	HT1	37.2	0.2	37.2	37
XC6	HT2	37		37	
XC6	HT3	36.8		36.8	
XC6	CK1	45.6	0.461880215	45.6	45.066667
XC6	CK2	44.8		44.8	
XC6	CK3	44.8		44.8	
Varieties	Treatment	Grains numbers per panicle	STDEV		
XC31	HT+Spm1	44.2	0.4163332	44.2	44.333333
XC31	HT+Spm2	44		44	
XC31	HT+Spm3	44.8		44.8	
XC31	HT+Spd1	42	0.115470054	42	42.066667
XC31	HT+Spd2	42		42	
XC31	HT+Spd3	42.2		42.2	
XC31	HT1	35.4	0.4163332	35.4	35.866667
XC31	HT2	36		36	
XC31	HT3	36.2		36.2	
XC31	CK1	48	0.305505046	48	48.333333
XC31	CK2	48.4		48.4	
XC31	CK3	48.6		48.6	

2018

Varieties	Treatment	Weight per panicle	STDEV		
XC6	HT+Spm1	2.04	0.004	2.04	2.044
XC6	HT+Spm2	2.044		2.044	
XC6	HT+Spm3	2.048		2.048	
XC6	HT+Spd1	1.794	0.012055428	1.794	1.7953333
XC6	HT+Spd2	1.808		1.808	
XC6	HT+Spd3	1.784		1.784	
XC6	HT1	1.738	0.017320508	1.738	1.718
XC6	HT2	1.708		1.708	
XC6	HT3	1.708		1.708	
XC6	CK1	2.332	0.035907288	2.332	2.2913333
XC6	CK2	2.278		2.278	
XC6	CK3	2.264		2.264	
Varieties	Treatment	Weight per panicle	STDEV		
XC31	HT+Spm1	1.834	0.03	1.834	1.834
XC31	HT+Spm2	1.804		1.804	
XC31	HT+Spm3	1.864		1.864	
XC31	HT+Spd1	1.872	0.005291503	1.872	1.866
XC31	HT+Spd2	1.864		1.864	
XC31	HT+Spd3	1.862		1.862	
XC31	HT1	1.398	0.003464102	1.398	1.396

XC31	HT2	1.398		1.398	
XC31	HT3	1.392		1.392	
XC31	CK1	1.95	0.086725621	1.95	2.0493333
XC31	CK2	2.088		2.088	
XC31	CK3	2.11		2.11	

2018

Varieties	Treatment	Thousand grains weigh	STDEV		
XC6	HT+Spm1	42.39	0.149777613	42.39	42.513333
XC6	HT+Spm2	42.47		42.47	
XC6	HT+Spm3	42.68		42.68	
XC6	HT+Spd1	41.89	0.370989667	41.89	41.813333
XC6	HT+Spd2	41.41		41.41	
XC6	HT+Spd3	42.14		42.14	
XC6	HT1	40.09	0.290516781	40.09	39.81
XC6	HT2	39.83		39.83	
XC6	HT3	39.51		39.51	
XC6	CK1	46.15	0.197315314	46.15	45.923333
XC6	CK2	45.83		45.83	
XC6	CK3	45.79		45.79	
Varieties	Treatment	Thousand grains weigh	STDEV		
XC31	HT+Spm1	43.06	0.125830574	43.06	43.076667
XC31	HT+Spm2	42.96		42.96	
XC31	HT+Spm3	43.21		43.21	
XC31	HT+Spd1	43.59	0.395769293	43.59	43.766667
XC31	HT+Spd2	43.49		43.49	
XC31	HT+Spd3	44.22		44.22	
XC31	HT1	39.12	0.339460356	39.12	39.183333
XC31	HT2	38.88		38.88	
XC31	HT3	39.55		39.55	
XC31	CK1	45.85	0.249466097	45.85	46.036667
XC31	CK2	46.32		46.32	
XC31	CK3	45.94		45.94	

2019

Varieties	Treatment	Grains numbers per panicle	STDEV		
XC6	CK1	46.2	0.757187779	46.2	47.066667
XC6	CK2	47.6		47.6	
XC6	CK3	47.4		47.4	
XC6	HT1	40	0.611010093	40	39.333333
XC6	HT2	38.8		38.8	
XC6	HT3	39.2		39.2	
XC6	CK+Spm1	47	0.8326664	47	47.933333
XC6	CK+Spm2	48.2		48.2	
XC6	CK+Spm3	48.6		48.6	
XC6	CK+Spd1	47.8	0.2	47.8	47.6
XC6	CK+Spd2	47.6		47.6	
XC6	CK+Spd3	47.4		47.4	

XC6	HT+Spm1	41.4	0.871779789	41.4	41.8
XC6	HT+Spm2	41.2		41.2	
XC6	HT+Spm3	42.8		42.8	
XC6	HT+Spd1	42.4	0.577350269	42.4	41.733333
XC6	HT+Spd2	41.4		41.4	
XC6	HT+Spd3	41.4		41.4	
Varieties	Treatment	Grains numbers per panicle	STDEV		
XC31	CK1	42.6	0.4163332	42.6	43.066667
XC31	CK2	43.2		43.2	
XC31	CK3	43.4		43.4	
XC31	HT1	38.6	0.2	38.6	38.6
XC31	HT2	38.4		38.4	
XC31	HT3	38.8		38.8	
XC31	CK+Spm1	46	0.230940108	46	45.866667
XC31	CK+Spm2	46		46	
XC31	CK+Spm3	45.6		45.6	
XC31	CK+Spd1	43.6	0.115470054	43.6	43.733333
XC31	CK+Spd2	43.8		43.8	
XC31	CK+Spd3	43.8		43.8	
XC31	HT+Spm1	42	0.461880215	42	41.733333
XC31	HT+Spm2	41.2		41.2	
XC31	HT+Spm3	42		42	
XC31	HT+Spd1	42	0.4163332	42	41.666667
XC31	HT+Spd2	41.2		41.2	
XC31	HT+Spd3	41.8		41.8	

2019

Varieties	Treatment	Weight per panicle	STDEV		
XC6	CK1	2.356	0.013613719	2.356	2.3713333
XC6	CK2	2.382		2.382	
XC6	CK3	2.376		2.376	
XC6	HT1	1.876	0.016370706	1.876	1.894
XC6	HT2	1.898		1.898	
XC6	HT3	1.908		1.908	
XC6	CK+Spm1	2.51	0.012489996	2.51	2.52
XC6	CK+Spm2	2.534		2.534	
XC6	CK+Spm3	2.516		2.516	
XC6	CK+Spd1	2.466	0.009237604	2.466	2.4553333
XC6	CK+Spd2	2.45		2.45	
XC6	CK+Spd3	2.45		2.45	
XC6	HT+Spm1	2.216	0.041004065	2.216	2.2386667
XC6	HT+Spm2	2.214		2.214	
XC6	HT+Spm3	2.286		2.286	
XC6	HT+Spd1	2.134	0.021007935	2.134	2.1546667
XC6	HT+Spd2	2.154		2.154	
XC6	HT+Spd3	2.176		2.176	
Varieties	Treatment	Weight per panicle	STDEV		
XC31	CK1	2.036	0.036896251	2.036	2.0453333
XC31	CK2	2.014		2.014	
XC31	CK3	2.086		2.086	
XC31	HT1	1.442	0.005033223	1.442	1.4413333
XC31	HT2	1.446		1.446	

XC31	HT3	1.436		1.436	
XC31	CK+Spm1	2.252	0.02247962	2.252	2.2273333
XC31	CK+Spm2	2.208		2.208	
XC31	CK+Spm3	2.222		2.222	
XC31	CK+Spd1	2.256	0.088030298	2.256	2.1693333
XC31	CK+Spd2	2.172		2.172	
XC31	CK+Spd3	2.08		2.08	
XC31	HT+Spm1	1.822	0.004	1.822	1.818
XC31	HT+Spm2	1.818		1.818	
XC31	HT+Spm3	1.814		1.814	
XC31	HT+Spd1	1.842	0.101080826	1.842	1.7386667
XC31	HT+Spd2	1.734		1.734	
XC31	HT+Spd3	1.64		1.64	

2019

Varieties	Treatment	Thousand grains weigh	STDEV		
XC6	CK1	50.88	0.543721743	50.88	50.303333
XC6	CK2	49.8		49.8	
XC6	CK3	50.23		50.23	
XC6	HT1	45.24	0.151437556	45.24	45.173333
XC6	HT2	45.28		45.28	
XC6	HT3	45		45	
XC6	CK+Spm1	52.76	0.848547779	52.76	52.433333
XC6	CK+Spm2	53.07		53.07	
XC6	CK+Spm3	51.47		51.47	
XC6	CK+Spd1	50.82	0.241729877	50.82	50.596667
XC6	CK+Spd2	50.63		50.63	
XC6	CK+Spd3	50.34		50.34	
XC6	HT+Spm1	49.11	0.28618176	49.11	48.84
XC6	HT+Spm2	48.87		48.87	
XC6	HT+Spm3	48.54		48.54	
XC6	HT+Spd1	48.39	0.072111026	48.39	48.31
XC6	HT+Spd2	48.25		48.25	
XC6	HT+Spd3	48.29		48.29	
Varieties	Treatment	Thousand grains weigh	STDEV		
XC31	CK1	45.81	0.14525839	45.81	45.8
XC31	CK2	45.94		45.94	
XC31	CK3	45.65		45.65	
XC31	HT1	43.29	0.292631737	43.29	42.966667
XC31	HT2	42.72		42.72	
XC31	HT3	42.89		42.89	
XC31	CK+Spm1	45.89	0.304357246	45.89	46.113333
XC31	CK+Spm2	46.46		46.46	
XC31	CK+Spm3	45.99		45.99	
XC31	CK+Spd1	46.59	0.690386365	46.59	45.886667
XC31	CK+Spd2	45.21		45.21	
XC31	CK+Spd3	45.86		45.86	
XC31	HT+Spm1	44.1	0.726659021	44.1	43.896667
XC31	HT+Spm2	43.09		43.09	
XC31	HT+Spm3	44.5		44.5	
XC31	HT+Spd1	43.27	0.331511689	43.27	43.54
XC31	HT+Spd2	43.91		43.91	
XC31	HT+Spd3	43.44		43.44	

2018

Varieties	Treatment	Grains numbers per panicle	housand grains weigh	grain yield	
XC6	HT+Spm1	43.2	42.39	4669.6824	4697.7443
XC6	HT+Spm2	43.4	42.47	4700.1549	
XC6	HT+Spm3	43.4	42.68	4723.3956	26.937617
XC6	HT+Spd1	38.8	41.89	4144.5966	4158.3785
XC6	HT+Spd2	39	41.41	4118.2245	
XC6	HT+Spd3	39.2	42.14	4212.3144	48.535378
XC6	HT1	37.2	40.09	3802.9374	3756.1721
XC6	HT2	37	39.83	3757.9605	
XC6	HT3	36.8	39.51	3707.6184	47.684659
XC6	CK1	45.6	46.15	5366.322	5277.6636
XC6	CK2	44.8	45.83	5235.6192	
XC6	CK3	44.8	45.79	5231.0496	76.814414
XC31	HT+Spm1	44.2	43.06	4853.2926	4869.905
XC31	HT+Spm2	44	42.96	4820.112	
XC31	HT+Spm3	44.8	43.21	4936.3104	59.853955
XC31	HT+Spd1	42	43.59	4668.489	4694.9274
XC31	HT+Spd2	42	43.49	4657.779	
XC31	HT+Spd3	42.2	44.22	4758.5142	55.327542
XC31	HT1	35.4	39.12	3531.3624	3583.8023
XC31	HT2	36	38.88	3569.184	
XC31	HT3	36.2	39.55	3650.8605	61.075527
XC31	CK1	48	45.85	5612.04	5674.0662
XC31	CK2	48.4	46.32	5716.8144	
XC31	CK3	48.6	45.94	5693.3442	54.983176

2019

Varieties	Treatment	Grains numbers per panicle	housand grains weigh	grain yield	
XC6	CK1	46.2	50.88	5994.1728	6036.7323
XC6	CK2	47.6	49.8	6044.724	
XC6	CK3	47.4	50.23	6071.3001	39.179785
XC6	HT1	40	45.24	4614.48	4530.8944
XC6	HT2	38.8	45.28	4480.0032	
XC6	HT3	39.2	45	4498.2	72.956804
XC6	CK+Spm1	47	52.76	6323.286	6408.2656
XC6	CK+Spm2	48.2	53.07	6522.8337	
XC6	CK+Spm3	48.6	51.47	6378.6771	103.0118
XC6	CK+Spd1	47.8	50.82	6194.4498	6141.505
XC6	CK+Spd2	47.6	50.63	6145.4694	
XC6	CK+Spd3	47.4	50.34	6084.5958	55.034196
XC6	HT+Spm1	41.4	49.11	5184.5427	5205.4935
XC6	HT+Spm2	41.2	48.87	5134.2822	
XC6	HT+Spm3	42.8	48.54	5297.6556	83.67747
XC6	HT+Spd1	42.4	48.39	5231.9268	5141.2182
XC6	HT+Spd2	41.4	48.25	5093.7525	
XC6	HT+Spd3	41.4	48.29	5097.9753	78.584322
XC31	CK1	42.6	45.81	4976.3403	5029.7254

XC31	CK2	43.2	45.94	5060.7504	
XC31	CK3	43.4	45.65	5052.0855	46.435405
XC31	HT1	38.6	43.29	4261.0347	4229.2379
XC31	HT2	38.4	42.72	4183.1424	
XC31	HT3	38.8	42.89	4243.5366	40.867373
XC31	CK+Spm1	46	45.89	5382.897	5393.4574
XC31	CK+Spm2	46	46.46	5449.758	
XC31	CK+Spm3	45.6	45.99	5347.7172	51.833606
XC31	CK+Spd1	43.6	46.59	5179.8762	5117.1615
XC31	CK+Spd2	43.8	45.21	5049.5049	
XC31	CK+Spd3	43.8	45.86	5122.1034	65.325996
XC31	HT+Spm1	42	44.1	4723.11	4672.0318
XC31	HT+Spm2	41.2	43.09	4527.0354	
XC31	HT+Spm3	42	44.5	4765.95	127.38439
XC31	HT+Spd1	42	43.27	4634.217	4625.8904
XC31	HT+Spd2	41.2	43.91	4613.1846	
XC31	HT+Spd3	41.8	43.44	4630.2696	11.179155
